# Linear B-cell epitopes in BthTX-I, BthTX-II and BthA-I, phospholipase A_2's _from *Bothrops jararacussu *snake venom, recognized by therapeutically neutralizing commercial horse antivenom

**DOI:** 10.1186/1753-6561-8-S4-P51

**Published:** 2014-10-01

**Authors:** Salvatore De-Simone, Paloma Napoleão-Pêgo

**Affiliations:** 1 CDTS/FIOCRUZ, Rio de Janeiro, Rio de Janeiro,22640-180, Brazil

## Background

The benefits from treatment with antivenom sera are indubitable. However, the mechanism for toxin neutralization has not been completely elucidated. A mixture of anti-bothropic and anti-crotalic horse antivenom has been reported to be more effective in neutralizing the effects of *B. jararacussu *snake venom than anti-bothropic antivenom alone. This study determined which regions in the three PLA_2_s from *B.jararacussu *snake venom are bound by antibodies in tetravalent anti-bothropic and monovalent anti-crotalic commercial horse antivenom.

## Methods

The epitopes recognized by therapeutic horse antivenom sera in the three major PLA_2_s present in the venom of *B. jararacussu* , BthTX-I, BthTX-II and BthA-I, were mapped using the parallel Spot-synthesis strategy. Two peptide libraries were designed to more precisely define the epitopes recognized by anti-bothropic and/or anti-crotalic horse antivenom. Each consisted of 69 peptide sequences of fourteen amino acids each that overlapped by nine amino acids and covered the entire protein sequences of the three PLA_2_s. The oligomeric structure of PLA_2S _proteins were solved by X-ray crystallography and are available in the protein data bank. The sequences of fifty PLA_2_s were selected and grouped into three sub-groups. Shared amino acids sequence from the 12 epitopes recognized by the reaction between the *B. jararacussu *PLA_2_s and anti-crotalic/anti-bothropic horse antivenom were analyzed by a multiple sequence alignment.

## Results and conclusions

Mapping experiments of BthTX-I, BthTX-II and BthA-I using two small libraries of 69 peptides each revealed six major IgG-binding epitopes that were recognized by both anti-bothropic and anti-crotalic horse antivenom. Two epitopes in BthTX-I were only recognized by the anti-bothropic horse antivenom, while anti-crotalic horse antivenom recognized four unique epitopes across the three PLA_2_s. Our studies suggest that the harmful activities of the PLA_2_s present in the venom of *B. jararacussu *are neutralized by the combinatorial treatment with both antivenom sera through their complementary binding sites, which provides a wide coverage on the PLA_2_s. In conclusion, the peptide arrays formed directly onto cellulose membranes allowed the identification of the major antigenic determinants in the three most important PLA_2_s (BthTX-I, BthTX-II and BthA-I) isolated from *B. jararacussu *snake venom recognized by commercial anti-bothropic and anti-crotalic horse antivenom. The cross-reactive epitopes located in the Lys49-PLA_2_, the major protein of this venom, recognized two specific epitopes located in a region of the enzyme responsible for the myotoxic action, which contributes to the deleterious effects of snake venom. In addition, the ability of the anti-crotalic horse antivenom to neutralize the anticoagulant activity was most likely associated with the acidic Asp49-PLA_2_. This study provides proof that the mixture of anti-crotalic and anti-bothropic horse antivenom is qualitatively more effective in neutralizing the effects unleashed of *B. jararacussu *snakebite. Regions recognized by the protective antivenom sera are prime candidates for improved venom cocktails or a chimeric protein encoding the multiple epitopes to immunize animals as well as for designing future synthetic vaccines.
